# The clinical outcome of the reduction of the patellar inferior pole fracture with wire cerclage through a generated bone hole, in combination with patellar concentrator: a retrospective comparative study

**DOI:** 10.1186/s13018-022-03014-7

**Published:** 2022-02-21

**Authors:** Rong Chen, Hong Cao, Zhibo Sun, Liangbo Jiang, Xiangwei Li, Lin Zhao, Xinghui Liu

**Affiliations:** 1 Department of Traumatic Orthopedics, Renmin Hospital, Hubei University of Medicine, Shiyan, 442000 Hubei China; 2 Department of Anatomy, Hubei University of Medicine, No. 30 Renmin South Road, Maojian District, Shiyan, 442000 Hubei China

**Keywords:** Patellar inferior pole fracture, Patellar concentrator, Fracture reduction, Cerclage

## Abstract

**Objective:**

The patellar inferior pole fracture is typically comminuted. Hence, achieving firm fixation and early activity is highly challenging. In this article, we employed the method of wire cerclage through a generated bone hole to reduce the fracture. Our objective was to compare the clinical efficacy of patellar concentrator alone with a combination of cerclage and patellar concentrator in the treatment of patellar inferior pole fracture.

**Methods:**

We conducted a retrospective review of patients with patellar inferior pole fractures, who underwent patellar concentrator fixation only (the control group) or cerclage combined with patellar concentrator fixation (the experimental group), performed by a single surgeon, between July 2015 and October 2019. Our analysis included surgical indexes like7 aspects (fracture gap after operation, operation time, intra-operative blood loss, intra-operative number of C-arm fluoroscopies conducted, Insall–Salvati ratio calculated immediately after operation, initial range of motion on the 7th day after operation, and fracture healing time), as well as the Bostman score and complications recorded on 1-, 3-, 6-, and 12-month follow up post operation.

**Results:**

A total of 94 patients with patellar inferior pole fracture and a minimum 1-year follow up were recruited. Following operation, the control group had 33 (71.74%) patients with a fracture gap of 0–2 mm and 13 (28.26%) patients with a fracture gap greater than 2 mm (*P* = 0.002). Conversely, the experimental group had 46 (95.83%) patients with a fracture gap of 0–2 mm and 2 (4.17%) patients with a fracture gap greater than 2 mm (*P* = 0.002). Compared to the control group, the experimental group did not experience enhanced operation time or intra-operative blood loss (*P* = 0.811, *P* = 0.823). The Insall–Salvati ratio and initial range of motion in the experimental group were larger than the control group (*P* = 0.037, *P* = 0.000). Alternately, the number of intra-operative C-arm fluoroscopies conducted and fracture healing time of the experimental group were considerably less than the control group (*P* = 0.003, *P* = 0.000). Moreover, at 1-, 3-, 6-, and 12-month follow ups after operation, the Bostman scores of the experimental group were remarkably higher than the control group (*P* < 0.05). At 12 months post operation, 23 cases (50%) were classified as excellent, 22 cases (47.83%) were good, and 1 case (2.17%) was poor in the control group (*P* = 0.005). In the meantime, in the experimental group, 38 cases (79.17%) were deemed as excellent and 10 cases (20.83%) were good (*P* = 0.005). Lastly, complications were detected in 3 cases (6.52%; 1 case of internal fixation loss, 2 cases of hematoma) within the control group, and in 1 case(2.08%; marginal wound necrosis) within the experimental group. There was no wound infection, implant discomfort, or broken fixation in either group.

**Conclusion:**

Managing the patellar inferior pole fracture with wire cerclage through a generated bone hole is both simple and effective. Moreover, an additional step of patellar concentrator fixation facilitates early functional exercise, with satisfactory clinical outcome.

## Background

Patella is the largest sesamoid bone in the human body, the inferior pole of which connects to the patellar ligament. Patellar fractures account for 1% of total body fractures, whereas inferior pole fractures account for 9.3–22.4% of patellar fractures [1]. The inferior patellar pole fracture is typically comminuted, with low bone mass, thereby making it is difficult to fix [2, 3]. Generally, upon fracture, the knee extension device is destroyed, which results in limited knee joint movement. As a result, surgical intervention is required.

At present, two major surgical procedures are available for the correction of inferior pole fractures. The first method removes the comminuted inferior pole of the patella and reconstructs the insertion point of the patellar ligament via a suture anchor or drilling hole. But this method often leads to bone defect, high tension within the patellar ligament, dislocation of the patella femoral joint, and so on [4]. Therefore, most scholars prefer an alternative method of correction, which utilizes patellar concentrator, steel wire, Kirschner wire, basket plate, and other materials to reduce and fix the fracture of the inferior pole of the patella [1, 3, 5–9]. There are multiple fixation methods, each with its unique advantages and disadvantages. However, there are certain common complications that need to be resolved. For example, internal fixation loosening, internal fixation cutting, difficult fixation of the comminuted bone mass, symptomatic implants, and so on [10, 11].

Although the anchor suture technique can be used to repair the inferior patellar fracture, it often requires auxiliary external fixation and early rehabilitation to be effective [3, 7, 8]. In addition, compared with Kirschner wire tension band technology, non-metallic fixation technology has the same clinical effect [12, 13], but a long-hinged knee brace was required for 3 weeks after operation [14]. Kirschner wire tension band is a common method of fixing patellar fractures. However, in comminuted fractures of the lower pole of the patella, this may lead to further displacement of the fracture, particularly, while inserting the Kirschner wires from the inferior pole. Compared to the Kirschner wire tension band, patellar concentrator has less symptomatic implants. Therefore, patellar concentrator can be effectively used to fix patellar inferior pole fracture, whilst permitting early rehabilitation, with a low incidence of post-operative complications [10]. In other works, scholars reported that separate vertical wiring can be used to fix the inferior patellar fracture. However, this may lead to wire cutting, particularly, in elderly patients with osteoporosis, which may lead to fixation failure [15]. Several scholars also used the percutaneous cerclage technique to reduce and fix the lower patellar pole fractures, with good outcomes, however, one case experienced wire slippage [16].

Based on the above findings, we employed the technique of wire cerclage through a generated bone hole to reset the inferior patellar pole fracture, and combine this technique with the patellar concentrator in the experimental group, whereas the control group only received patellar concentrator. The purpose of this study was to retrospectively analyze the surgery-related indexes and clinical outcomes of the two afore-mentioned fixation methods. Our hypothesis was that, compared to the patellar concentrator only technique, the wire cerclage through a generated bone hole, along with the patellar concentrator, will achieve a more satisfactory fracture reduction and produce a more enhanced clinical outcome.

## Materials and methods

### Patient selection

**Fig.1:**
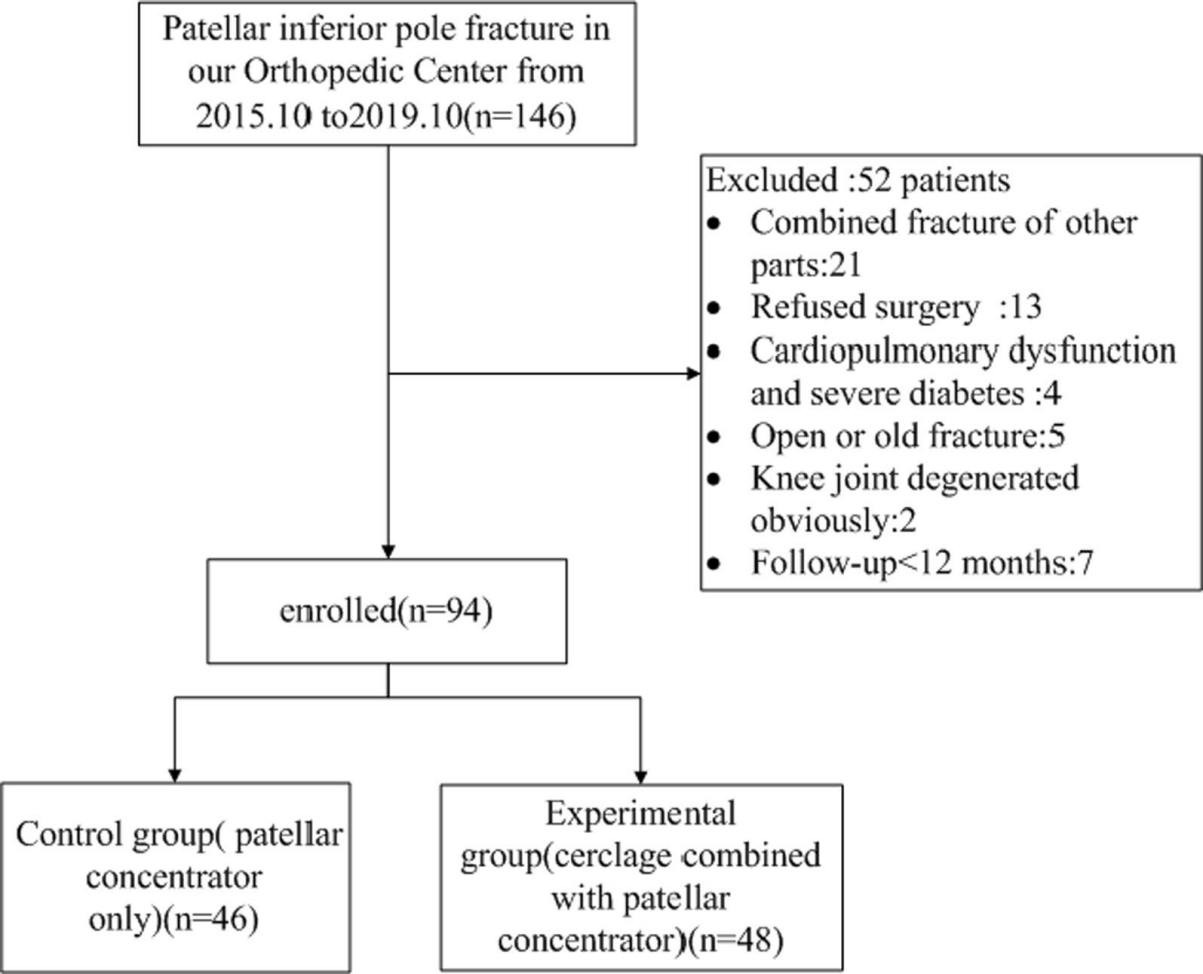
Study flow chart

This study protocol was approved by the Ethics Committee of Renmin Hospital affiliated with the Hubei University of Medicine. The inclusion criteria were as follows: X-ray or CT examination depicting unilateral patellar inferior pole fracture, patients aged 30–80 years old, patients who either received only patellar concentrator fixation or cerclage combined with patellar concentrator fixation, performed by the same surgeon, with minimum of 1-year follow-up, patients who provided signed informed consent forms and who cooperated with medical staff to complete relevant diagnosis and treatment protocols. The exclusion criteria included: patients with pathological fracture, open or old fracture, pyogenic knee arthritis, obvious degeneration, other knee injuries, or fracture in other areas, cardiopulmonary dysfunction, severe diabetes, and patients who refused surgery. Between July 2015 and October 2019, 94 patients with patellar inferior pole fractures were recruited for analysis (Fig. 1).

### Surgical technique

Upon successful combined spinal epidural anesthesia, patients were placed in the supine position, and tourniquets were applied intraoperatively with 300–337.5 mmHg pressure. In each case, an anterior midline patellar approach was adopted with exposure from the quadriceps tendon down to the patellar tendon. Special attention was given to avoid excessive damage to the surrounding fascia whilst exposing the fracture position.

Control group: The lower part of the patellar concentrator was initially inserted close to the inferior pole of the patella. Next, the quadriceps femoral tendon was cut close to the upper edge of the patella with a sharp knife and the upper region of the patellar concentrator was inserted. Subsequently, forceps were used to reduce the fracture and place two screws in the upper and lower regions of the patellar concentrator. The reduction quality and patellar concentrator positioning were then examined via C-arm fluoroscopy. Lastly, fracture fixation stability was confirmed by flexing the knee at least 90 degrees post operation.

**Fig. 2:**
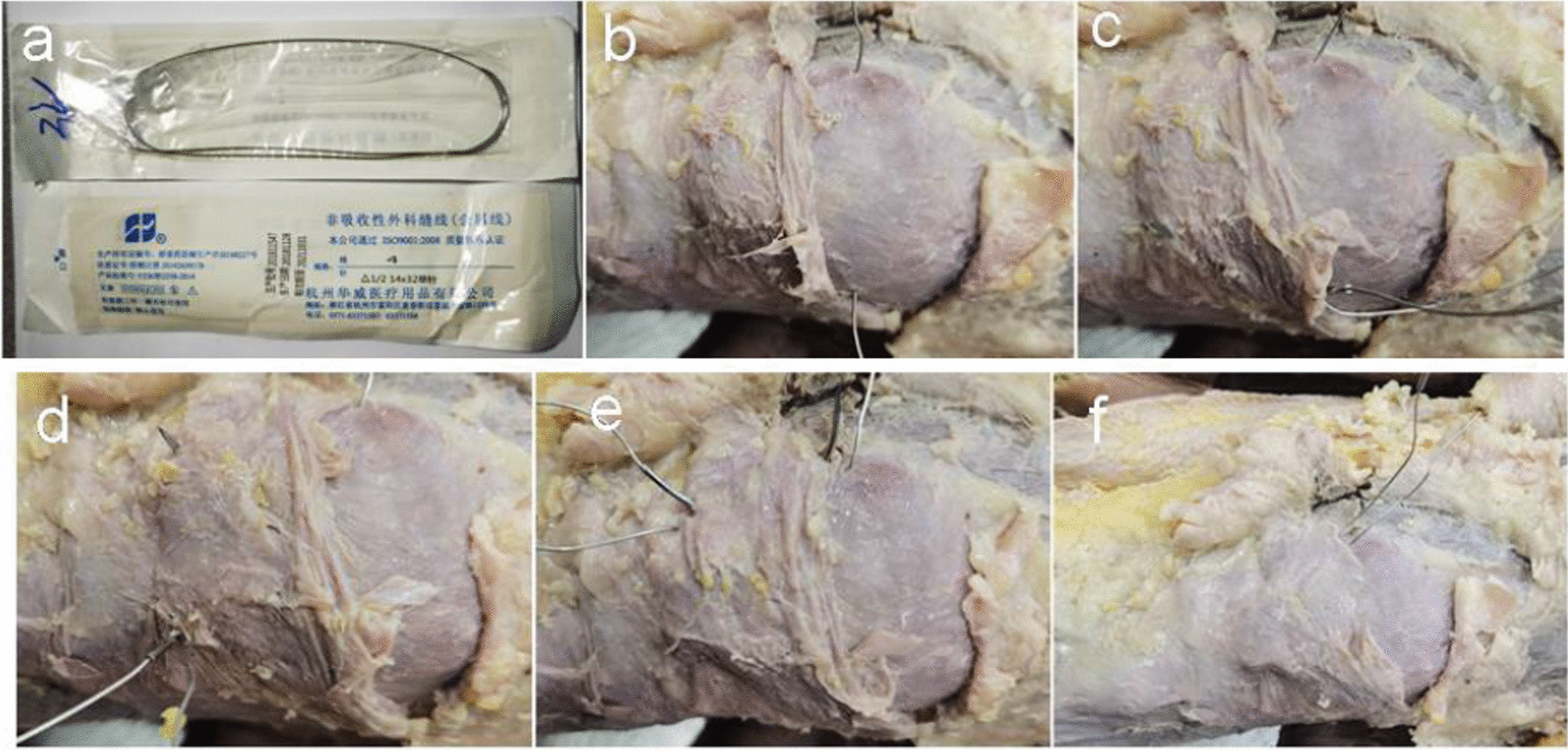
Demonstration of the procedure of suture and cerclage with metal wire passing through a generated bone hole on a corpse **a** Wire with suture needle **b** Bone hole drilled with a 2.0 mm diameter Kirschner wire and the metal wire was inserted **c** Piercing of the first needle under the Parapatellar ligament close to the bone surface. **d** Piercing of the second needle under the patellar tendon. **e** Placing the third needle in the same position as the first needle. **f** Tightening the metal wire with close attention to the fracture reduction

Experimental group: We first drilled a hole and placed a 2.0 mm diameter Kirschner wire in the proximal patella. Next, a wire with a suturing needle was penetrated through the bone hole (Fig. 2b), and suturing was used to penetrate around the patella (Fig. 2c–e). Of note, the distal patella was also penetrated under the patellar tendon (Fig. 2d). Finally, the steel wire was slowly tightened at the knee extension position, with special attention to the corresponding location across from the fracture end (Fig. 2f). The subsequent patellar concentrator process followed the same procedure as mentioned in the control group.

### Post-operative management and follow-up

Following anesthesia, we performed both ankle pump exercises and quadriceps isometric contraction training. Ice compress was provided within 48 h of operation. There was no need to fix the knee joint, and the knee joint was able to flex without external aid on the third day post operation. On postoperative day 5–7, the patients were discharged from the hospital and were provided guidance regarding knee flexion and quadriceps strength exercises outside the hospital. The patients were followed up every 2 weeks within 3 months of operation, and once a month after that.

### Outcome measures

Both groups of patients were compared in terms of surgical indexes that included 7 aspects, namely, fracture gap after operation, operation time, intra-operative blood loss, number of intra-operative C-arm fluoroscopies conducted, Insall–Salvati ratio (Lateral radiographs were performed to measure the patellar height using the Insall–Salvati ratio, which was defined as the ratio of the lengths of the patellar tendon to the longest diagonal line of the patella) calculated immediately after the operation, initial range of motion on the 7th day after operation, and fracture healing time.

The Bostman score and post-surgical complications were also recorded at the 1-, 3-, 6-, and 12-month follow ups after operation. The clinical Bostman grading scales were employed for the evaluation of knee function using 8 aspects listed as follows: range of movement (0–6 points), knee pain (0–6 points), routine work (0–4 points), muscle atrophy (0–4 points), assisted walking(0–4 points), effusion (0–2 points), giving way (0–2 points), and stair-climbing (0–2 points). Based on these scales, 28–30 points were deemed as excellent, 20–27 points were good, and less than 20 points were regarded as poor.

### Statistical analysis

All data were analyzed using the SPSS version 25.0 statistical software. The counting data are presented as mean ± standard derivation. Measurement data are expressed as %, and comparison of rates were done with the χ^2^ test. Inter-group comparison adopted the t-test, whereas, comparison at different time points adopted the repeated measurement analysis of variance. Differences were considered significant when *P* value was < 0.05.

## Results

### General data of patients in the control and experimental groups

Overall, 94 patients with patellar inferior pole fracture and a minimum of 1-year follow-up were enrolled between July 2015 and October 2019. This included 46 patients in the control group and 48 patients in the experimental group (Fig. 1). Both groups exhibited comparable general demographics (Table 1).

**Table 1:** Comparison of general demographics within the control and experimental patient cohorts (mean ± SD; *n*, %)

	Control group	Experimental group	*χ*2/*t*	*P*
Gender			0.260	0.794
Male	17 (36.96)	19 (39.58)		
Female	29 (63.04)	29 (60.42)		
Age (years)	56.02 ± 10.00	55.81 ± 11.24	0.095	0.924
Body mass index (kg/cm2)	22.23 ± 1.32	22.14 ± 1.21	0.349	0.728
AO/OTA Type			1.592	0.111
A1	18 (39.13)	16 (33.33)		
A2	9 (19.57)	5 (10.42)		
C1.3	19 (41.30)	27 (56.25)		
Cause of fracture			0.760	0.448
Fall injury	34 (73.91)	33 (68.75)		
Traffic accider	10 (21.74)	14 (29.17)		
Other	2 (4.35)	1 (2.08)		
Affected side			0.824	0.412
Left	25 (54.35)	22 (45.83)		
Rights	21 (45.65)	26 (54.17)		
Injury time before operation (days)	2.02 ± 0.80	2.27 ± 0.82	1.489	0.140

### Comparing the reduction outcomes between the control and experimental groups

**Fig. 3:**
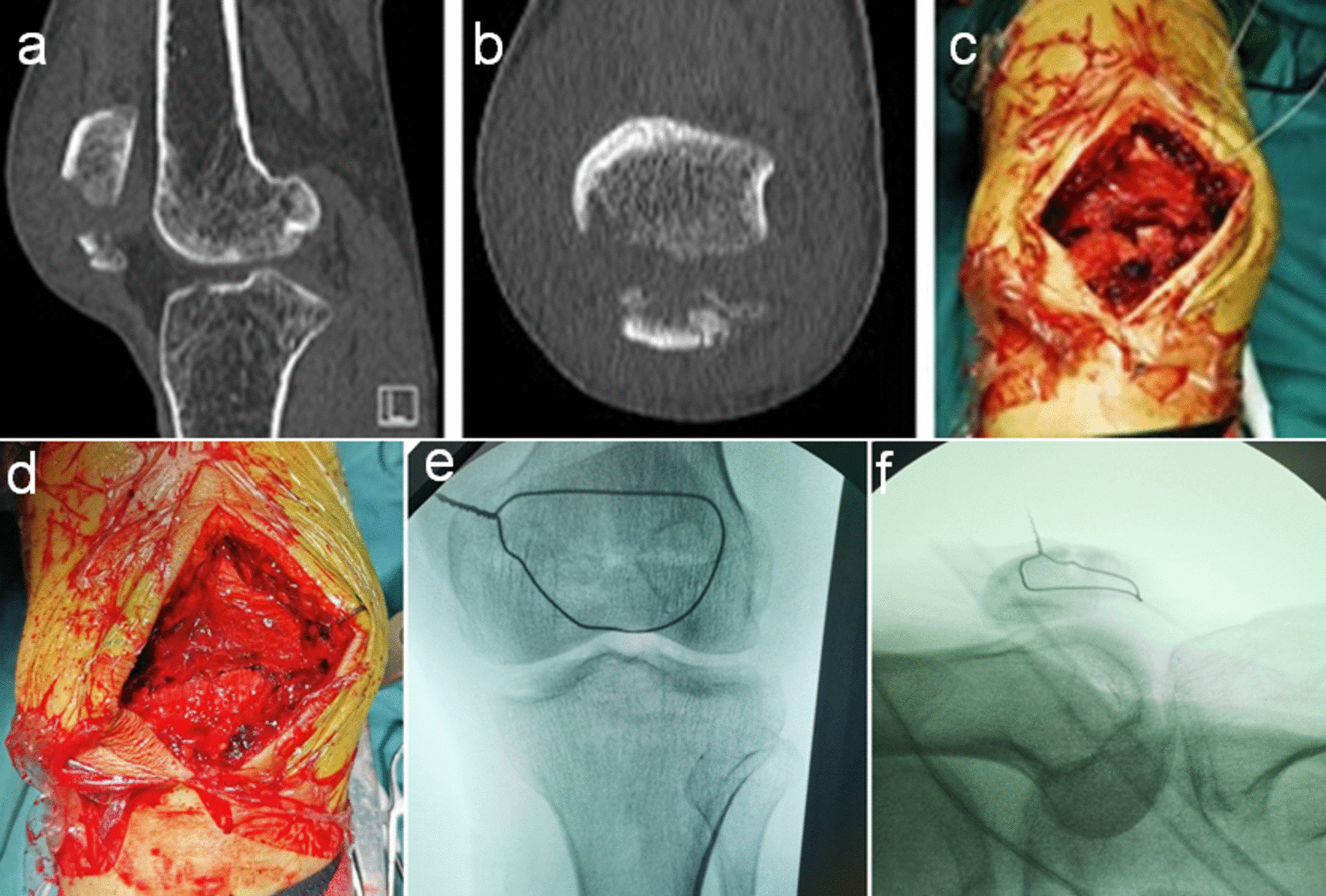
Intra-operative reduction **a–b** Pre-operative CT examination. **c** Intra-operative images captured before reduction. **d** Intra-operative images captured after reduction **e–f** Intra-operative C-arm fluoroscopy after reduction

**Fig. 4:**
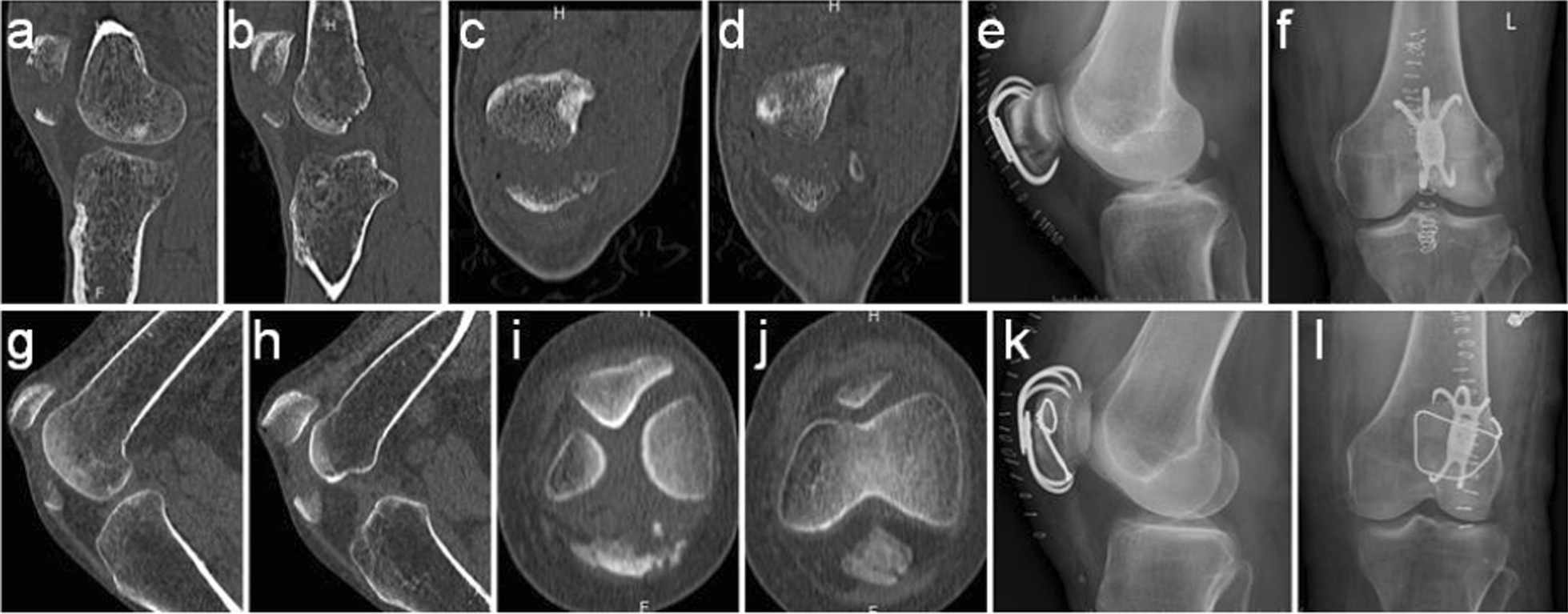
Comparison of the control and experimental patient groups before and after reduction **a–f** A 38 year old male was admitted to the hospital for correction of fracture caused by a fall 3 h prior to admission **a–b** Pre-operative CT examination of the sagittal plane. **c–d** Pre-operative CT examination of the coronal plane. **e–f** Post-operative X-ray. **g–l** A 65 year old female was admitted to the hospital for correction of fracture caused by a fall 5 hourspriorto admission. **g-h** Pre-operative CT examination of the sagittal plane. **i–j** Pre-operative CT examination of the coronal plane. **k–l** Post-operative X-ray

The process of wire cerclage through a generated bone hole involved five steps (Fig. 2b–f). First, a metal wire (Fig. 2a) was used to needle three times to get around the patella (Fig. 2c–e). Figure 3a and b illustrates a typical comminuted fracture of the inferior patella, as evidenced by pre-operative imaging. Upon tightening of the steel wire, the comminuted bone block was successfully reduced under direct vision, and the steel wire did not slip (Fig. 3c–f). Figure 4a–f depicts a case of comminuted fracture of the inferior pole of the patella, which was treated with conventional reduction and patellar concentrator fixation (a case from the control group). Obvious fracture gap was detected on the post-operative X-ray (Fig. 4e). However, in another case, reset was done with wire cerclage (Fig. 4g–l) (a case from the experimental group), and no obvious fracture gap was observed on the post-operative X-ray (Fig. 4k). In all, the control group had 33 (71.74%) cases with fracture gap of 0–2 mm and 13(28.26%) cases with fracture gap larger than 2 mm, while the experimental group had 46(95.83%) cases with fracture gap of 0–2 mm and only 2(4.17%)cases with fracture gap larger than 2 mm. There was statistical significance between the two patient cohorts (*P* = 0.002).

### Comparing surgical indexes between the control and experimental groups

**Fig. 5:**
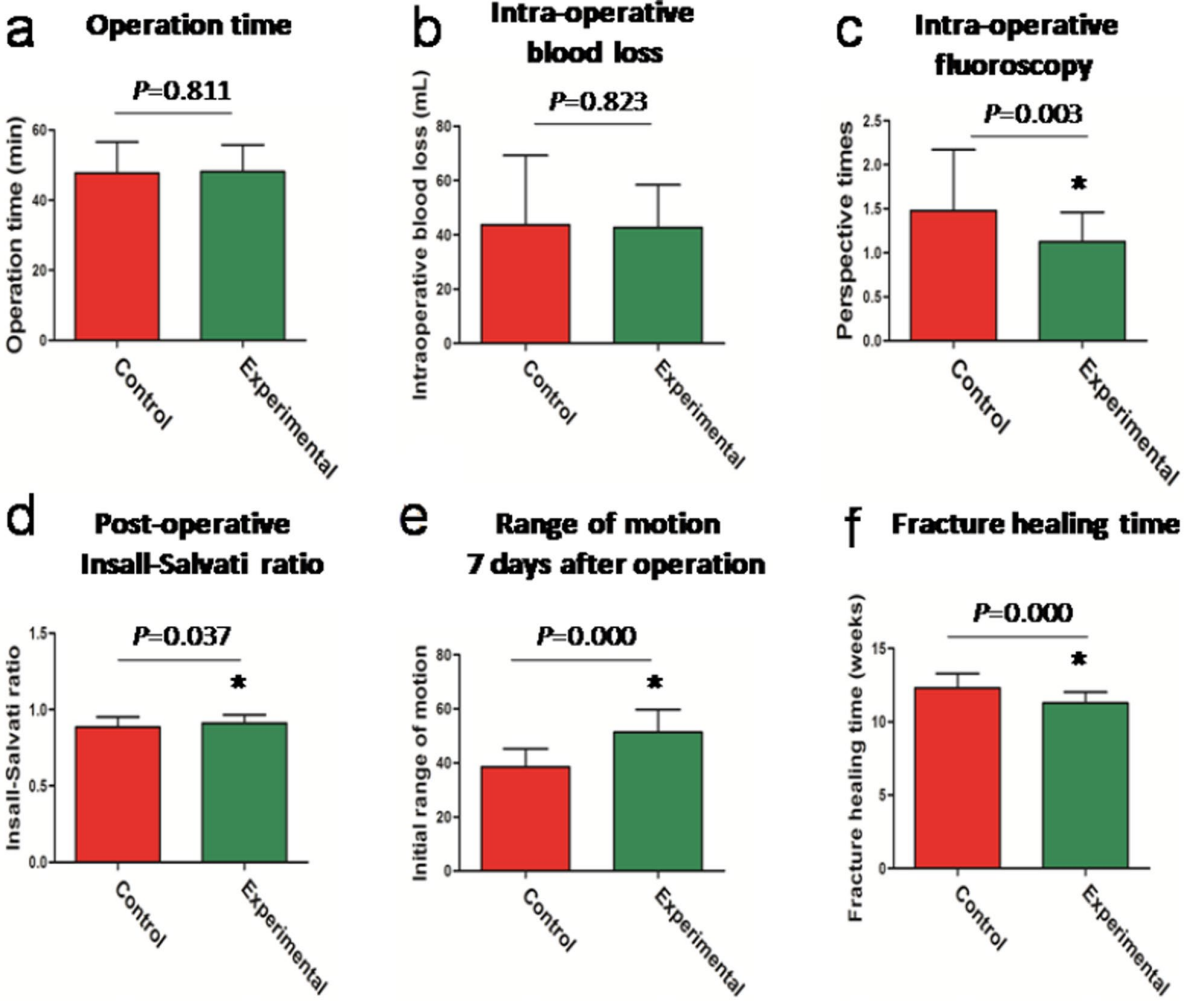
Comparison of the surgical indexes between the control and experimental patient cohorts **a.** Operation time **b.** Intra-operative blood loss **c.** Number of intra-operative C-arm fluoroscopies conducted **d.** Insall–Salvati ratio calculated immediately after the operation **e.** Initial range of motion on the 7th day post operation **f.** Fracture healing time.*P < 0.05 compared to the control group

**Fig. 6:**
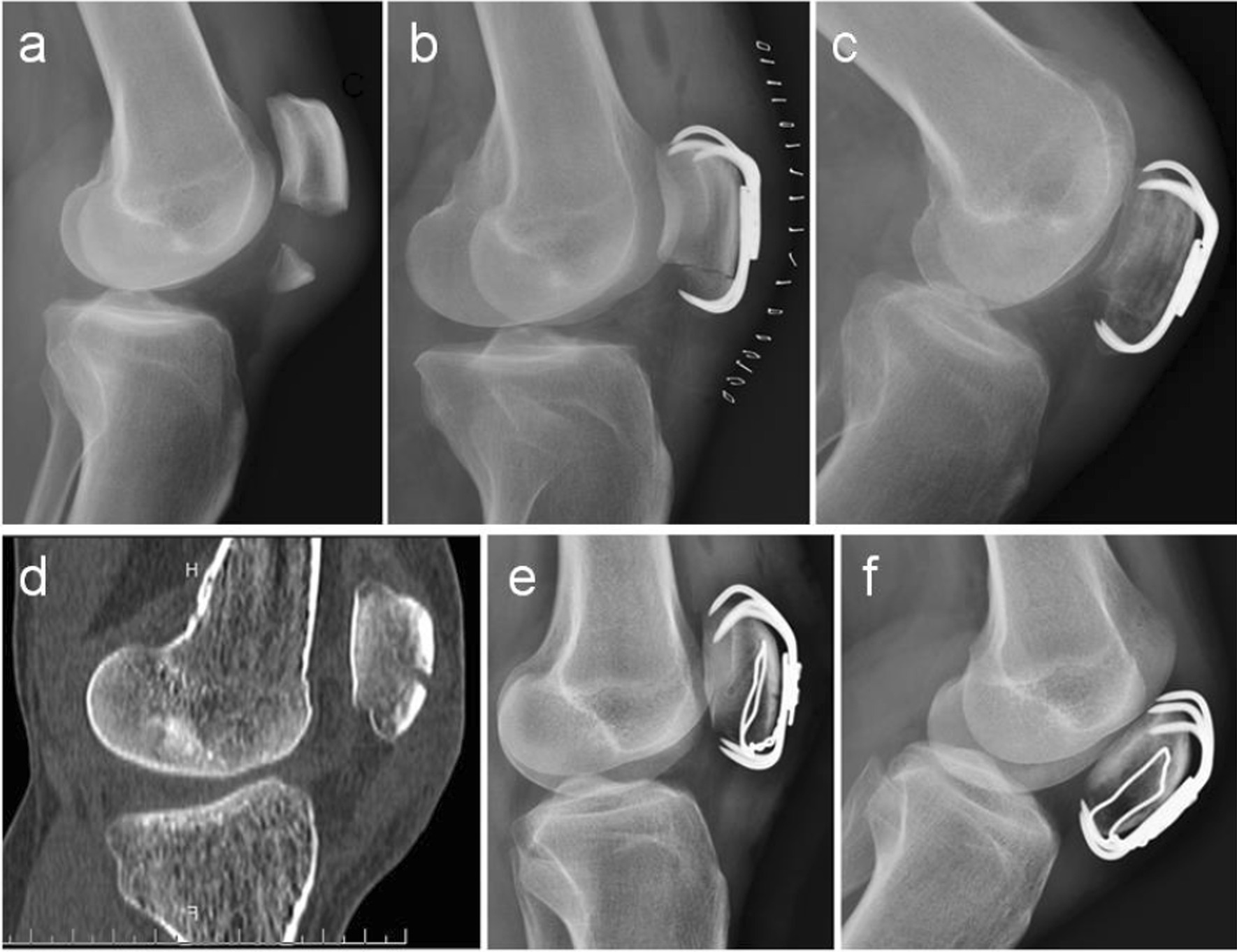
Comparison of postoperative fracture healing between the control and experimental patient cohorts **a-d.** A 42 year old male was admitted to the hospital for correction of fracture caused by a fall 4 h prior to admission. **a.** Pre-operative X-ray lateral film **b.** Post-operative X-ray lateral film **c.** Post-operative X-ray lateral film at the 12-week follow up **d-f.** A 38 year old female was admitted to the hospital for correction of fracture caused by a fall 3 h prior to admission. **d.** Pre-operative CT examination of the sagittal plane **e.** Post-operative X-ray lateral film **f.** Post-operative X-ray lateral film at the 12-week follow up

Compared to the control patients, wire cerclage usage in the experimental group did not increase operation time or intra-operative blood loss (*P* = 0.811, *P* = 0.823) (Fig. 5a, b). However, intraoperative C-arm fluoroscopy was performed more frequently in the control group, relative to the experimental group (*P* = 0.003) (Fig. 5c). The experimental group exhibited a larger Insall–Salvati ratio, compared to the control group (*P* = 0.037) (Figs. 5d, 6b, e). On the 7th day post operation, the initial range of motion in the experimental group was remarkably higher than the control group (*P* = 0.000) (Fig. 5e). In contrast, the fracture healing time of the experimental group was drastically shorter than in the control group (*P* = 0.000) (Figs. 5f, 6a–f).

### Bostman score and postsurgical complications

**Fig. 7:**
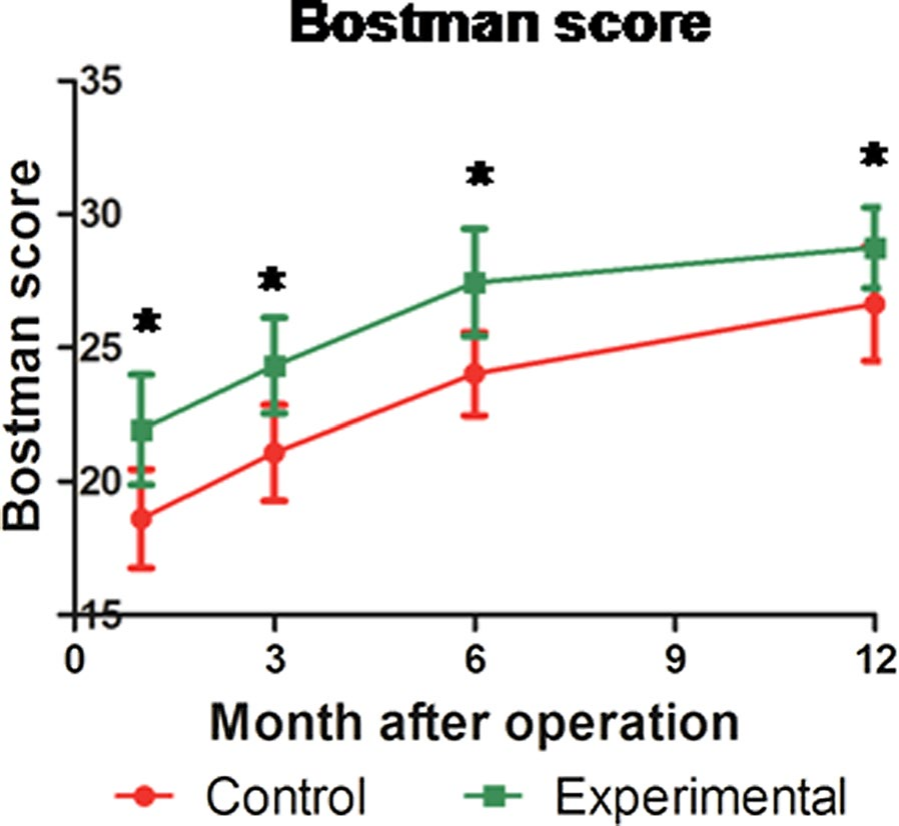
Differences in Bostman scores between the control and experimental groups at different time points post operation. The Bostman score of both groups gradually increased over time, according to the repeated measurement analysis of variance.*P < 0.05 compared to the control group

The Bostman scores of both control and experimental groups gradually increased over time, as evidenced by the scores calculated at the 1-, 3-, 6-, and 12-month follow ups post operation (*P* < 0.05). However, the Bostman score of the experimental group remained significantly higher than the control group (*P* < 0.05) (Fig. 7). At the 12-month follow up, 23(50%) patients from the control group achieved an excellent score, 22(47.83%) patients received good, and 1(2.17%) received a poor score. In the experimental group, however, 38(79.17%) patients received excellent scores, 10(20.83%) received good, and none received a poor score. There was statistical significance between both groups (*P* = 0.005). Complications occurred in 3 cases (6.52%; 1 case of internal fixation loss, 2 cases of established hematoma)in the control group, and in 1 case (2.08%; marginal wound necrosis) in the experimental group. The patient experiencing loss of internal fixation refused a repeat operation and was treated with plaster external fixation. There was no wound infection, implant discomfort, or broken fixation in either group.

## Discussion

The inferior patellar pole fracture is often comminuted, and the small bone mass is difficult to fix. Fortunately, the comminuted bone block connects to the patellar ligament, so it can be reduced via anchor suture technology [3, 7, 8]. However, the suture is elastic and requires additional protection via external fixation after operation. Additionally, there is a great risk in early exercise. Hence, to ensure early exercise after patellar fracture surgery, one must follow the principle of tension band fixation, which states that during exercise the muscle contraction converts tension to power of axial compression. The Kirschner wire tension band fixation technique achieves excellent outcomes in the treatment of patellar fractures, however, patients often complain of implant discomfort [17]. Therefore, some scholars use the screw tension band fixation method instead [15]. However, it is not easy to implant the Kirschner wire or screw into the patella in appropriate positions [18]. This, in turn, aggravates the displacement of the patellar inferior pole fracture. Fortunately, using the patellar concentrator abrogates the need to drill holes in the patella, which avoids the problem of fracture displacement caused by Kirschner wire or screw implantation. In addition, the design of the patellar concentrator conforms to the principle of the tension band, which places continuous pressure on the fracture end [19]. Hence, the patellar concentrator possesses advantages of both simple operation and early functional exercise, and is suitable for various types of patellar fractures [5, 10]. However, our results revealed that the patellar concentrator alone produces poor reduction of the inferior patellar pole fracture. Lateral comminuted fractures were earlier shown to be difficult to fix with patellar concentrator alone [20], so multiple C-arm fluoroscopies are needed to ensure proper reduction. In addition, repeated reduction may negatively affect blood supply to the fracture site and thereby prolong healing time.

At present, the reported wire cerclage fixation is commonly used as an auxiliary fixation procedure for other types of fracture fixation [21]. In a prior report, an author successfully used percutaneous steel wire cerclage fixation technique to reduce and fix an inferior patellar pole fracture [16]. Moreover, another scholar employed modified cerclage wiring to correct comminuted patellar fracture, with satisfactory results [20]. Given these evidences, it was likely that the steel wire cerclage method can be used for reducing and fixing patellar fractures. Since the traditional wire cerclage method involves the steel wire passing under the quadriceps tendon and encompassing the patella, the wire can easily slip and cause fixation failure [16]. To avoid steel wire sliding and unstable fixation, we generated a bone hole for the wire to provide a fixed fulcrum. In addition, we employed the suture method to guide the patellar wire cerclage, which made it easy to operate and saved operation time. Compared to the control group, the experimental group had comparable operation time and intra-operative blood loss, and conducted relatively less C-arm fluoroscopies to confirm proper fixation. Additionally, unlike single wire cerclage, the multiple vertical wire cerclage provided ample strength to protect the knee joint from early exercises [15]. Furthermore, separate vertical wiring combined with the Krackow suture [6] or combined with rim-plate [22] was reported to provide secure fixation and favorable clinical outcomes. However, in the process of penetrating vertical wires, the risk of fracture displacement cannot be avoided. Moreover, although plates are often used for reduction and fixation of comminuted fractures, plates placed at the starting point of the patellar ligament can cause significant discomfort [9]. Particularly, in elderly patients with osteoporosis, fixation failure may be caused by wire cutting problems [15]. Therefore, after wire cerclage reduction, we used patellar concentrator fixation to protect the knee joint from early exercise damage. This not only prevented fracture displacement caused by perforating Kirschner wire or screw at the patella fracture, but also significantly reduced stress cutting caused by the direct contact of the patellar concentrator with patella. Good reduction and firm fixation are the premise of early rehabilitation exercise [1, 23]. Based on our analysis, the initial range of motion of our experimental group was markedly enhanced, compared to the control group.

Based on earlier reports, the normal range of InsallSalvati ratio is 0.8–1.2, and less than 0.8 represents patella baja [24]. The degree of patellar fracture reduction may affect the longest diagonal of the patella, and, therefore, the InsallSalvati ratio. In a previous report, suture bridge anchorage decreased the InsallSalvati ratio, with no patella-femoral pain and limited range of motion [3]. In our study, the InsallSalvati ratio was within normal range after operation in both the control and experimental groups. However, the InsallSalvati ratio was slightly higher and closer to the normal value in the experimental group. The Bostman score is commonly used to evaluate the clinical efficacy of patellar fracture fixations. In a study that treated patellar inferior pole fracture with a mini-plate usage and tension band, the average Bostman score was 28.1 at the 1 year follow up post operation [1]. Additionally, in a study comparing a Novel Tension Band and Patellotibial Tubercle Cerclage, the average Bostman scores were 28.5 and 25.8, respectively [23]. In our study, the average Bostman score of the experimental group was 27.44 and 28.75 at the 6-and 12-month follow ups, respectively.

We encountered certain limitations in our study, namely, limited case numbers and short follow-up duration. Hence, more cases from multiple trauma centers and results from long-term follow up are needed to verify our conclusions. In the future, we plan to use this reduction technique for other types of patellar fractures.

## Conclusion

Patellar inferior pole fracture can be easily reset with a simple wire cerclage through a generated bone hole. However, special caution is necessary to avoid stripping excess soft tissue around the fracture to prevent comminuted bone block displacement and augment difficulty of reduction. Compared to the patellar concentrator alone, the procedure of steel wire cerclage combined with patellar concentrator achieves a more satisfactory reduction of the patellar inferior pole fracture, with reliable fixation, early functional exercise, and fast fracture healing.

## Data Availability

The datasets generated and analyzed during the current study are available from the corresponding author on reasonable request.
